# The Use of Optical Coherence Tomography to Demonstrate Dark and Light Adaptation in a Live Moth

**DOI:** 10.1093/ee/nvac044

**Published:** 2022-06-28

**Authors:** Simon Berry

**Affiliations:** Simon Berry Optometrist, The Jam Pot, 41 Marshall Terrace, Gilesgate, Durham DH1 2HX, England, UK

**Keywords:** Lepidoptera, superposition compound eye, light adaptation, pigment migration, moth

## Abstract

To work effectively, the eyes of nocturnal insects have a problem they must overcome. During the night, the light levels are low, so their eyes need to be very sensitive; but they also need a way of adapting to environmental light conditions, and protecting those sensitive organs, if a bright light is encountered. Human eyes have a pupil that changes size to regulate light input to the eye. Moths (Lepidoptera) use a light absorbing pigment that moves position to limit the light within the eye. This pigment migration is difficult to record because it is a dynamic process and will only occur in a live moth. This paper presents the first use of Ocular Coherence Tomography as a method of viewing anatomical detail in a compound eye. This is noninvasive and does not harm the insect. To demonstrate the effectiveness, this article documents the dynamic process of light adaptation within a moth’s eye.

The compound eye is the most common type of eye design found on our planet (Land 2014). It is found in arthropods such as insects and crustaceans. Compound eye structure can be broadly classified as being either an apposition or superposition eye depending on the structure of the eye and the image it produces.

Many species of moth and other nocturnal insects possess a superposition compound eye (Exner 1891, Land and Nilsson 2012) (Fig. 1). This type of eye is well suited to low light levels as it is very sensitive to light (Warrant 2001, Lees and Zilli 2019).

**Fig. 1.**
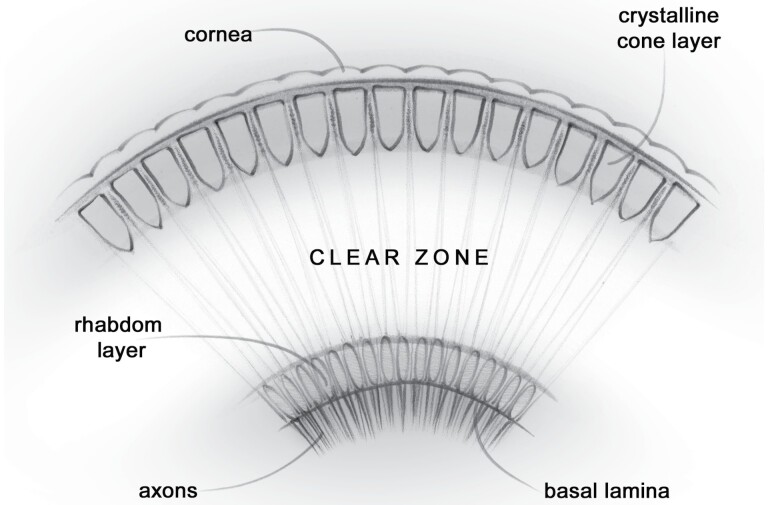
The anatomy of a superposition compound eye. Figure illustrator – Juliet Percival.

To allow the insect to adapt to environmental light conditions nocturnal moths light-adapt (Bernhard 1964, Post and Goldsmith 1965, (Walcott 1969). They manage this process in a number of ways, one of which is by moving a light absorbing pigment into an area of the eye where it can restrict the amount of light reaching the sensitive rhabdom layer. This longitudinal pigment migration occurs between the crystalline cone layer and the clear zone (Post and Goldsmith 1965, Warrant and McIntyre 1996).

When an insect is dark adapted, pigment is squeezed in between the crystalline cones. This allows the maximum possible light to pass through the clear zone and to the rhabdom layer beneath. When the insect becomes light adapted the pigment migrates into the clear zone, thereby reducing the amount of light reaching the rhabdoms (Post and Goldsmith 1965, Warrant and McIntyre 1996) (Fig. 2).

**Fig. 2.**
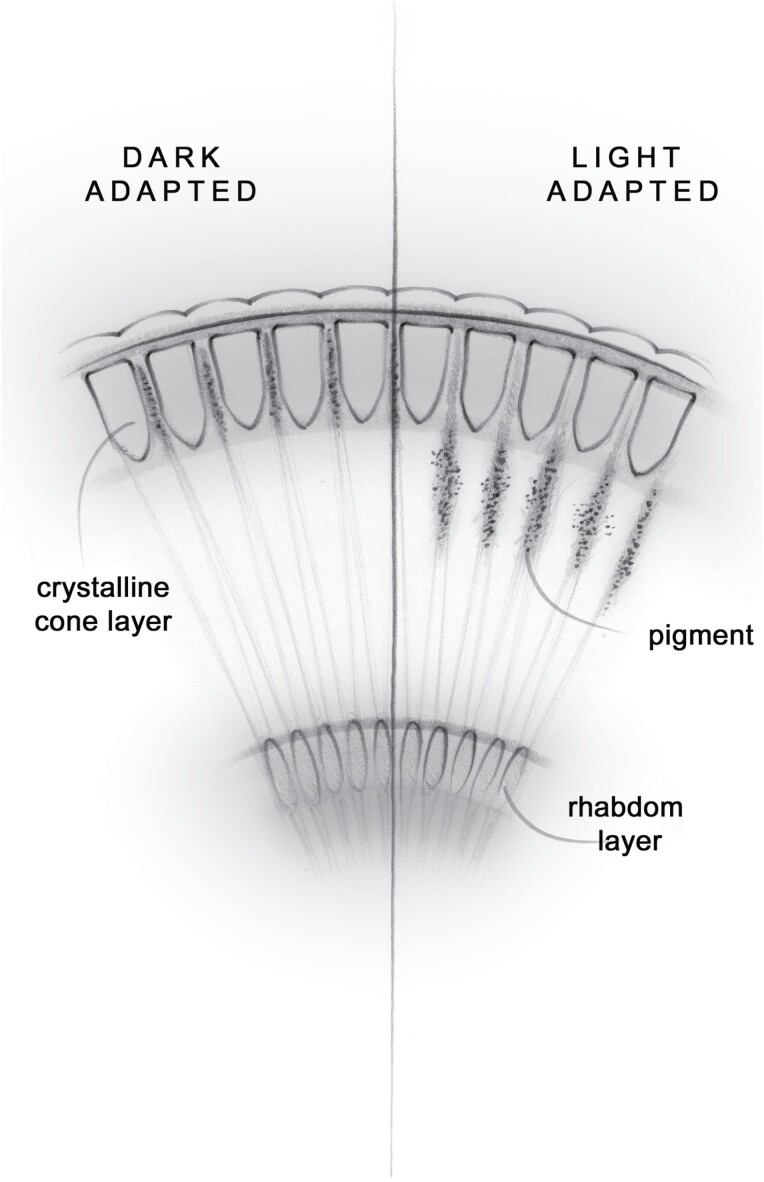
Adaptation in a superposition compound eye showing pigment migration that occurs during light adaptation. Figure illustrator – Juliet Percival.

In the case of moths, it is possible to gauge the state of adaptation of the insect by observing its ‘eye glow’. This is the reflected light from the insect’s tapetum, a reflective layer of cells beneath the rhabdom layer. (Collins 1934, Land and Nilsson 2012). The light that creates the ‘eye glow’ has to pass through the clear zone, so the more pigment that has migrated into with the clear zone, the less obvious and bright the eye glow is (Kunze 1969).

The process of light adaptation in moths is well understood from previous articles that have studied eye glow (e.g., Day 1941, Post and Goldsmith 1965). Other articles have correlated and quantified the amount of pigment migration within the clear zone and the effect it has on eye glow (e.g., Dreisig 1981, Höglund 1963).

When describing how pigment migrates into the clear zone, many articles rely on diagrams, or microscopic slides showing the pigment migration to help the reader visualize the effect that pigment has on the adaptation state (Post and Goldsmith 1965, Warrant and McIntyre 1996, Land and Nilsson 2012, Satoh et al. 2017). Dark and light adaptation is a dynamic process. By necessity, any microscopic examination of the eye requires dissection of a dead insect and will show a snapshot of the adaptive state at that point in time.

This article describes how Optical Coherence Tomography (OCT) can be used to show the pigment migration during adaptation in real time. The approach allows visualization of the process of light adaptation in a novel way. The advantage of the technique is that it is noninvasive and can be performed multiple times on a live insect. This allows real-time scans as the pigment migrates into the clear zone of the superposition eye.

OCT technology is used for the noninvasive optical imaging of biological tissue. It is often compared to medical ultrasound because the principles involved are somewhat similar (one uses sound and the other uses light). OCT is widely used in Ophthalmology to obtain cross-sectional information of structures within the eye. It can present these data in either 2-dimensional or 3-dimensional scans. It uses the principle of low-coherence light interferometry to capture information, typically with a near-infrared light source. It can gain information about any structure that the light can penetrate through before it is absorbed. (Podoleanu 2012, Aumann et al. 2019).

This article presents OCT scans that demonstrate pigment migration, along with scans of moth superposition eyes that show both dark and light adaptive states.

To demonstrate that scans are truly documenting the adaptation process, both dark and light adaptive states are compared. The method described is also repeated on a small skipper butterfly. This butterfly is known to possess a superposition eye but does not light adapt by the same longitudinal pigment migration as nocturnal moths do, (Horridge et al. 1972), and hence can be used as a control.

This article aims to demonstrate the usefulness of Ocular Coherence Tomography in entomology. It can be used to visualize the structures and processes within the compound eye of a live insect.

## Method

The moths were caught using a Heath Moth Trap. No particular type of moth was sought after and various light sources were used. The moths were selected based on what was caught that particular night, and which species had already been scanned. The small skipper butterfly was caught using a butterfly net.

The insects were caught between May and August 2021 in Durham, England within 2 miles of the Optometry Practice where the OCT scanner was held. They were identified with help from the Collins Field Guide to Moths of Great Britain and Ireland (Waring and Townsend 2009).

All insects were caught, transported, and scanned using 125 ml specimen pots made from transparent polypropylene (purchased from NHBS Ltd, UK). Since the insects were all alive, the scan was performed through the walls of the specimen pot. The pots varied in quality and whilst there is some degradation of the image because of this, it does not detract from the results of the scans or affect the interpretation of the scans. (A less optically clear pot would generate more general noise within the scan, but not affect the image of the biological structures shown).

The OCT scanner used was a Topcon 2000. This is a second generation spectral-domain OCT. The anterior scans of the machine were modified to obtain the highest possible interpolation of 32 scans. (This means that 32 scans were averaged to produce a result. While this produces a higher resolution image, in a dynamic situation an averaging scan may result in loss of detail). A combination of 3 mm, 6 mm, radial and 3D scans were used with the best quality scans being included in this article.

The insects were initially dark adapted. They were kept in a dark bag for at least an hour. The first scan was completed with the room in darkness to try and ensure the insect stayed dark adapted.

The lights of the room were then turned on and various scans were taken as the insect became light adapted. A directional white LED light source was used for this purpose (Microscope Illuminator made by GT Vision Ltd with a color temperature of around 6500 K). A light meter was used to measure the light levels (Digital Lux Meter BT-881D, UK), however the recorded light levels are only intended as an illustrative guide to the conditions during the method. The light level meter reading for these stages were:

Testing Room with lights on: 200 lux
Testing room with lights off: 1.2 lux
Directional LED: 1,500 lux

Attempts were made to try and scan the dark adapted and light adapted eye from the same angle, however this was not always possible if the insect moved. All line scans were horizontal and taken as close to the center of the eye as possible.

The insects were released back into the wild in the place they were originally caught.

Scans were downloaded as .tiff files and opened in Adobe Lightroom. The only manipulation of the original image was the contrast levels or cropping. The images were exported as .jpegs to be included in this article.

## Results

**Fig. 3.**
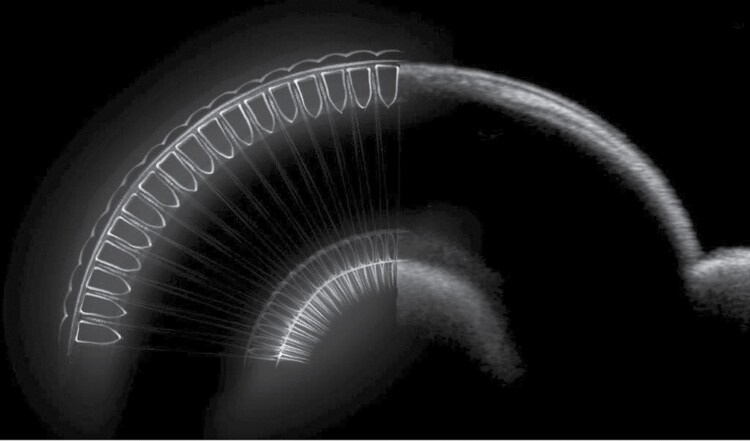
A live OCT image presented beside a diagram of the corresponding anatomy of a moths eye. Cells depicted are not to scale and are used for illustrative purposes only. Figure illustrator – Juliet Percival.

A live OCT image of a moth’s eye is presented beside a diagram of the corresponding anatomy of the moth’s eye in Fig. 3 to help interpret the subsequent OCT scans.

### Comparison of Dark and Light Adapted Superposition Eyes

**Fig. 4.**
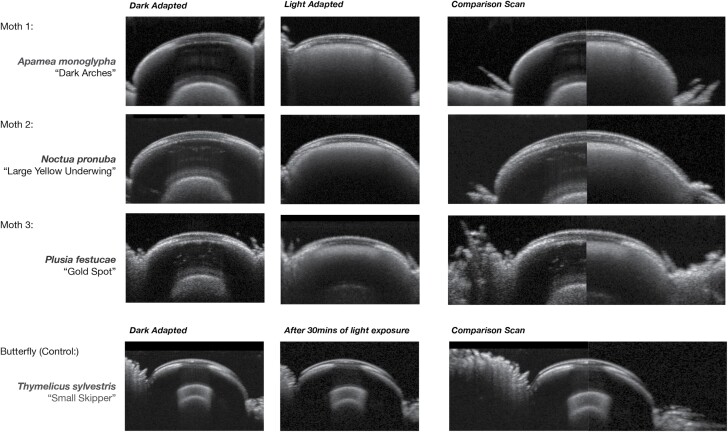
OCT scans demonstrating dark and light adaptive states.

The scans for light and dark superposition eyes (Fig. 4) show that the OCT technology can indeed document light and dark adaptation of a live insect. Eyes were compared for three different species of moth. The composition of the clear zone is different in the different adaptive states.

In the dark adapted state, the clear zone is optically clear. Light from the OCT passes through and shows no detail. Light penetrates through to the rhabdom layer and resolves structural information from this layer. In the light adapted state, the pigment has migrated into the clear zone. The light from the OCT is scattered and reflected by the pigment and the composition of the clear zone is changed. The light does not penetrate completely through the clear zone so the detail from the rhabdom layer beneath is lost.

As a control, the superposition eye of the skipper butterfly was also scanned. The results demonstrate that despite being exposed to light for over 30 min, there are no differences in the OCT scans and no difference in the composition of the clear zone.

### Transitional Phases Between Light and Dark Superposition Eyes

**Fig. 5.**
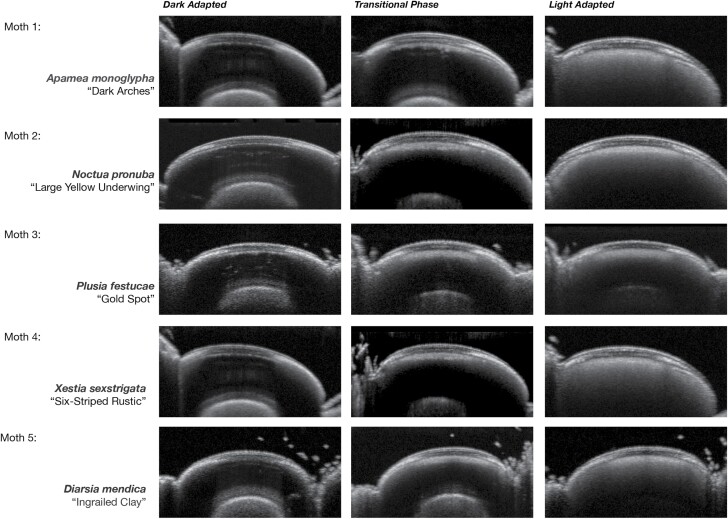
OCT scans demonstrating transition stages in light adaption.

Scans during the transitional phase that occurs within the clear zone during light adaptation are presented in Fig. 5. Five different species of moth are used to demonstrate this. As the insect becomes light adapted and more pigment migrates into the clear zone, the rhabdom layer beneath becomes less visible.

Light adaptation is a dynamic process. To further visualize this process two time-lapse videos were produced, which were challenging due to the movement of live moths Supp Videos (online only). These were difficult to put together since all moths are live and move around. The time-lapse has been arranged to focus on the back of the crystalline cone layer, since this is where the pigment migration occurs.

## Discussion

The superposition compound eye is found in most nocturnal insects because it is more sensitive in low light levels than an apposition compound eye. (Warrant 2001 , Land and Nilsson 2012). It is also found in deep water crustaceans (Land and Nilsson 2012). There are numerous excellent articles describing the optics of compound eyes. (e.g., Shaw 1969, Land and Nilsson 2012) This article does not dwell on the optics but rather the physical characteristics when the eye is either dark or light adapted and the visualization of the eye’s structure.

Despite the assumption that superposition eyes are only useful for nocturnal insects, some diurnal insects do also possess a superposition compound eye, the skipper butterfly being the example included in this article (Horridge et al. 1972). Both moths and butterflies belong to the order Lepidoptera and are very closely related.

The skipper butterfly was selected because it is an example of a diurnal superposition eye that does not light adapt in the same way as the nocturnal moth superposition eye does. The scan of the skipper eye can therefore be used as a control for the absence of the migratory pigment as the light levels change. It demonstrates there is no artefact induced in the method used.

The OCT scan shows the pigment migration that occurs during light adaptation, but it is possible to determine whether an insect is light or dark adapted without directly observing this pigment migration. Most moths possess a tapetum. This is a reflective layer beneath the rhabdom layer. The purpose of the tapetum is to reflect the light back through the light sensitive layer. (Collins 1934, Land and Nilsson 2012). This enables two light passes through the rhabdom layer instead of one and increases the chance of photon capture by the light sensitive cells. This is why eyes that possess a tapetum are more sensitive in low light levels.

When the insect is dark adapted, the tapetum is visible as a glowing disc referred to as eye glow (Collins 1934, Land and Nilsson 2012). This is visible when the eye is illuminated and viewed at roughly the same angle. As the eye becomes light adapted, and pigment migrates into the clear zone, the eye glow reduces and in some cases is not visible at all. In this way the eye glow can be used to determine the state of adaptation of the eye.

**Fig. 6.**
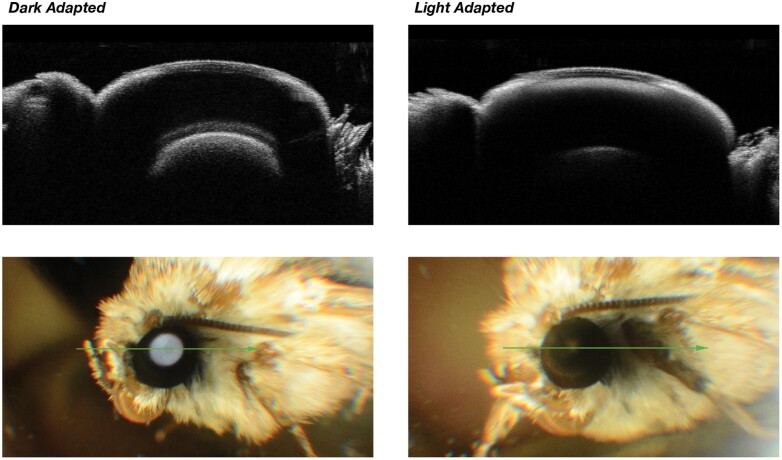
Correlation of OCT scans to moth's eye glow for dark and light adapted eyes.

The Topcon 2000 also takes a photographic image when the OCT scan is taken. This is only meant as an orientation image, but because the flash from the camera is at the same angle it is possible to see the eye glow, or lack of eye glow, from the tapetum and in this way the state of dark or light adaptation can be confirmed (Fig. 6). In this way, the OCT scans can be correlated with the eye glow to confirm the state of adaptation. All the scans in this paper have been checked in this way.

The Topcon 2000 uses a near infra-red light with a wavelength of 850 nm. It is capable of picking up information about any structure that the light can penetrate through. The information it picks up is limited to how deep the light can penetrate before it is fully absorbed. Certain structures and pigments absorb more of the light and influence the amount of detail seen.

The adaptation state of the insect also influences how much detail of the deeper rhabdom layer can be seen. As the pigment migrates into the clear zone, more of the near infra-red light from the OCT is absorbed by the pigment, it cannot penetrate as deeply into the tissue and so less detail is seen.

As can be seen from the scans, the rhabdom layer is more visible in a dark adapted eye than a light adapted eye. This also demonstrates how effective the moths light adaptation is.

## Proposed Future Work

The purpose of this article is to show that OCT technology is capable of documenting light adaptation in a superposition compound eye. The fact that the insect is alive, and the adaptation is seen in real time, means that many aspects of the process can be further explored in greater detail.

For example, it is interesting to note that much work has been done on the circadian rhythm of insects and the possible interruptions from nocturnal artificial light sources (Bradshaw and Holzapfel 2010, Gaston et al. 2017). It is possible that the light adaptive state of the insect caused by artificial lights may influence its behavior, or form part of the trigger for the circadian rhythm.

This initial article is demonstrating how OCT technology can be used in insect research. It is beyond the scope of this article to comment fully on the biological characteristics of the light adaptation process itself. Although it is worth noting that for the moths scanned in this article, the time taken for light adaptation to occur is consistent with other papers written on the subject. (Collins 1934, Warrant et al. 1996). It takes around 30 min for a moth’s eye to fully change their state of adaptation.

The physiological adaptation process to light is relatively slow, and during this period the insect’s perception is not optimized for the environmental light levels. For example, if a light source causes an insect to light adapt and then that light source is taken away, it will take a period of time for the insect to be become dark adapted and see effectively in low light levels. Equally, if the insect flies from the light source they may not be able to perceive the same level of detail in lower light levels until they have become dark adapted again.

From the OCT scans, it appears that the beginning of the pigment migration is not instantaneous. There is a short delay before the pigment migration becomes visible. This may be because it takes time for the pigment to migrate and show in the scan. However, there could possibly be a biological reason why this may occur. Having a lag before pigment migration means that if the insect encounters a brief flash of bright light, they may be able to recover quickly from it because the pigment migration has not started. They may not lose their fully dark adapted state immediately (as humans do) and are able to move around their environment more effectively.

Conversely, the time lag in transition from light to dark adaption may disadvantage moths with light adapted eyes for a time period if they move away from a light source into the dark. Further research is needed to determine whether the state of light adaption affects moth behavior.

**Fig. 7.**
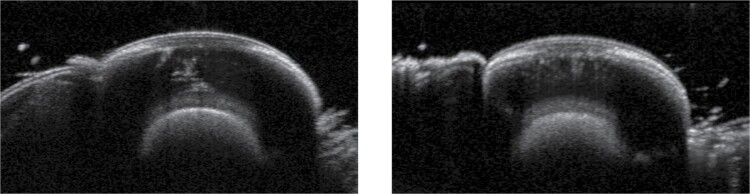
Showing a structure or process within the clear zone just before light adaption.

The OCT may also be able to investigate the biological mechanisms of the pigment migration. Some of the scans pick up a process happening within the clear zone just before the pigment migration from the crystalline cones (Fig. 7). The resolution of the scan is not enough to interpret exactly what process is occurring, it could potentially show a clump of light absorbing pigment deposited into the clear zone as part of the adaptation process. It is anticipated that future work may indeed shed some light into what is happening in this process (for example by using swept source OCT scanners).

This article has concentrated on light adaptation, because the process has never been able to be visualized in this way before, but the technique can be used for other measurements. It is possible to measure characteristics of the eye such as radius of curvature, and depth measurements that may lead to better understanding of the optics of the eye.

## Conclusions

Ocular Computerized Tomography a useful tool to visualize structures within the compound eye of a live insect. This paper presents the first scans documenting the pigment migration in a superposition eye in real time, and in a live insect.

## Supplementary Material

nvac044_suppl_Supplementary_GoldSpot

nvac044_suppl_Supplementary_YellowUnderwing
