# Correction to: The Use of Optical Coherence Tomography to Demonstrate Dark and Light Adaptation in a Live Moth

**DOI:** 10.1093/ee/nvac105

**Published:** 2022-12-09

This is a correction to: Simon Berry, The Use of Optical Coherence Tomography to Demonstrate Dark and Light Adaptation in a Live Moth, *Environmental Entomology*, Volume 51, Issue 4, August 2022, Pages 643–648, https://doi.org/10.1093/ee/nvac044

In the originally published version of this manuscript, supplementary video files were not included.

Publisher sincerely regrets this error which has now been corrected online.

